# Erbium-Doped Lu_2_O_3_-MgO and Sc_2_O_3_-MgO IR-Transparent Composite Ceramics

**DOI:** 10.3390/nano13101620

**Published:** 2023-05-12

**Authors:** Dmitry Permin, Alexander Belyaev, Vitaliy Koshkin, Sergey Kurashkin, Stanislav Balabanov, Ksenia Smetanina, Maxim Boldin, Oksana Klyusik

**Affiliations:** 1 Faculty of Chemistry, N.I. Lobachevsky Nizhny Novgorod State University, 23 Gagarin Ave., 603022 Nizhny Novgorod, Russia; svk_doma@mail.ru (S.K.); smetanina@nifti.unn.ru (K.S.); boldin@nifti.unn.ru (M.B.); oks.kluysik@yandex.ru (O.K.); 2 G.G. Devyatykh Institute of Chemistry of High-Purity Substances of the Russian Academy of Sciences, 49 Tropinin Street, 603137 Nizhny Novgorod, Russia; belyaev@ihps-nnov.ru (A.B.); koshkin@ihps-nnov.ru (V.K.); balabanov@ihps-nnov.ru (S.B.)

**Keywords:** SHS, nanopowders, composites, optical ceramics, hot pressing

## Abstract

Novel IR-transparent ceramics of erbium-doped Lu_2_O_3_-MgO and Sc_2_O_3_-MgO composites have been successfully obtained using a combination of glycine–nitrate self-propagating high-temperature synthesis and vacuum hot-pressing methods. Composites have densities greater than 99.5% of those calculated by X-ray diffraction and consist of uniformly distributed submicron grains of magnesium and rare earth oxides. The transmittances of 1.5 mm thick composites are as high as 84.5% and 78.9% at ~5 µm for Er:Lu_2_O_3_-MgO and Er:Sc_2_O_3_-MgO, respectively. Both composites are favorable matrices for doping with erbium ions, which exhibit intense luminescence in the visible, near, and mid-IR under relevant excitation. The position of the luminescence bands is similar to Er:Lu_2_O_3_ and Er:Sc_2_O_3_ ceramics; the lifetimes of the ^4^I_13/2_ state are 8.85 ± 0.1 ms and 5.7 ± 0.2 ms for 3%Er:Lu_2_O_3_-MgO and 3%Er:Sc_2_O_3_-MgO, respectively.

## 1. Introduction

The development of optical ceramic technology has led to significant progress in the production of new laser materials (active media, saturable absorbers, magneto-optical media, etc.) and contributed to the advancement of laser technology [1,2]. Typically, laser ceramics are single-phase and have a cubic crystal lattice to avoid passive optical losses due to scattering [3]. Recently, it has been shown that it is possible to obtain two-phase Y_2_O_3_-MgO [4,5,6,7] and Gd_2_O_3_-MgO [8,9,10] ceramics with transmission in the wavelength range of 1.5–8 μm at the level of single crystals of rare earth sesquioxides by reducing the grain size to at least a tenth of a wavelength, e.g., 100–200 nm. Such nanocomposites have advantages over sesquioxides in both mechanical properties and thermal conductivity [11,12]. These properties are important for active media, and it is attractive to use composites as a matrix for doping with ions whose luminescence bands fall within the corresponding transparency range.

However, there are just a few studies of Y_2_O_3_-MgO ceramics doped with Er^3+^ or Ho^3+^ ions for laser applications. Safronova et al. [13] obtained Ho^3+^:Y_2_O_3_-MgO composites and, based on the absorption cross section at 1931 nm (σ_abs_ = 0.51 × 10^−20^ cm^2^) and the luminescence cross section at 2118 nm (σ_em_ = 0.29 × 10^−20^ cm^2^), showed that the composite can be a promising material for high-power eye-safe lasers operating in the 2 μm wavelength range. Later, Wang et al. [14] found that Ho^3+^:Y_2_O_3_-MgO composites exhibit luminescence properties similar to those of Ho:Y_2_O_3_ single crystals and ceramics and have higher thermal conductivity.

Ma et al. [15] obtained Er^3+^:Y_2_O_3_-MgO ceramics with a short wavelength transmission edge (10% level) of less than 1 µm and a transmittance of up to 80% in the mid-infrared wavelength range. The authors state that the composite may be a promising candidate for use as an eye-safe laser host material at wavelengths of ~1.5 µm. Blair et al. [16] later carried out the detailed spectroscopic investigation of Er^3+^:Y_2_O_3_-MgO ceramics also with respect to ~1.5 µm laser applications.

Erbium ions have near-zero solubility in magnesia at composite fabrication temperatures and form solid solutions of Er_x_Y_2−x_O_3_, which is the reason for the similarity in luminescence characteristics of doped yttria ceramics or single crystals and composites. Like yttria, lutetia and scandia are very attractive matrices for doping with Er^3+^ ions [17,18], and their mutual solubility with magnesia decreases with decreasing temperature, causing them to form two-phase composites with MgO [19,20,21]. As far as we know, there is only one publication on the preparation of Lu_2_O_3_-MgO infrared material [22] and not one on Sc_2_O_3_-MgO transparent ceramics. This is probably due to the fact that the main driver for research on Y_2_O_3_-MgO and Gd_2_O_3_-MgO composites is their use as windows in the 2–5 µm range for aerospace applications. Lutetia and scandia are about two orders of magnitude more expensive than yttria, so Lu_2_O_3_-MgO or Sc_2_O_3_-MgO composites, with unknown operational advantages for such applications, do not appear promising. However, in the case of laser applications, the replacement of RE oxide (or even the use of their solid solutions) can adjust the luminescent properties of materials and improve the characteristics of laser materials. For example, dopant ions in scandia experience stronger crystal fields than in yttria, leading to larger total Stark splitting of their multiplets, allowing longer wavelength radiation. Lutetia, on the other hand, has a moderate reduction in thermal conductivity when heavily doped with Er-ions (which is typically required to achieve effective ~2.8 μm generation), allowing for increased power density. This potentially expands the use of composites in medical and environmental monitoring applications.

This work is devoted to the study of the possibility of obtaining IR-transparent Lu_2_O_3_-MgO and Sc_2_O_3_-MgO nanocomposite ceramics doped with erbium ions. The method of initial nanopowder synthesis, the effect of hot-pressing temperatures on the optical transparency and microstructure of the composites, their microhardness, fracture toughness, and luminescence spectra, on the radiative lifetimes of the ^4^I_13/2_ state Er^3+^ ions were investigated.

## 2. Materials and Methods

The composite ceramics of rare earth (RE) sesquioxides with magnesia were fabricated via hot pressing of nanopowders obtained using the method of self-propagating high-temperature synthesis (SHS). This approach has been shown to be effective in the fabrication of Y_2_O_3_-MgO [11] and Gd_2_O_3_-MgO [23] composites earlier.

The starting materials for the synthesis of SHS precursors were scandia, Sc_2_O_3_ (purity 99.99%, Polirit, Moscow, Russia); lutetia, Lu_2_O_3_ (99.99%, Polirit, Russia); erbia, Er_2_O_3_ (99.99%, Polirit, Russia); magnesia, MgO (99.99%, Unikhim, St.Petersburg, Russia); nitric acid, HNO_3_ (99.9999%, Khimreaktiv, N. Novgorod, Russia); and glycine, NH_2_CH_2_COOH (99.9%, Vitareaktiv, Dzerzhinsk, Russia).

Nitrate salts of scandium, lutetium, and magnesium were obtained by dissolving their oxides in nitric acid. Glycine was used as a reducing agent (fuel). According to the literature data [6,24], the optimal volume ratio of RE sesquioxides and magnesia in IR-transparent ceramics is 50:50%. This composition results in a dense microstructure of the ceramic material with the smallest grain size.

Solutions of RE and magnesium nitrates were mixed in a given ratio and then glycine was added based on the stoichiometry of the reactions:

6 RE(NO_3_)_3_ + 10 NH_2_CH_2_COOH → 3 RE_2_O_3_ + 20 CO_2_ + 25 H_2_O + 14 N_2_

9 Mg(NO_3_)_2_ + 10 NH_2_CH_2_COOH → 9 MgO + 20 CO_2_ + 25 H_2_O + 14 N_2_

The amount of erbium was chosen to replace 1 or 3% of lutetium or scandium atoms, respectively. Thus, the composition of the final material can be expressed by the formulas: (Sc_0.99_Er_0.01_)_2_O_3_-MgO, (Sc_0.97_Er_0.03_)_2_O_3_-MgO, (Lu_0.99_Er_0.01_)_2_O_3_-MgO, and (Lu_0.97_Er_0.03_)_2_O_3_-MgO.

The glycine–nitrate solution was evaporated at 110 °C and then, to initiate SHS, a quartz flask containing the precursor was placed in an oven preheated to 500 °C. As a result of combustion, a soft foam consisting of nanoscale RE_2_O_3_-MgO (RE = Lu, Sc, Er) particles was formed. The powders were then additionally air-annealed in a muffle furnace at 800 °C for 5 h.

The powders were compacted in a 15 mm diameter stainless-steel mold at a pressure of 40 MPa, then hot pressed in a graphite mold at temperatures of 1200–1450 °C and a uniaxial pressure of 50 MPa on home-made equipment. The compacts were insulated with graphite paper to reduce the interaction with the mold material. Heating was performed using graphite heaters at a rate of 25 °C/min; the residual pressure in the chamber did not exceed 10 Pa. The heating mode included a twenty-minute isothermal hold at 800 °C to desorb moisture and carbon dioxide, heating to sinter temperature, holding for 60 min, and free cooling. The initial uniaxial pressure on the compact was 3 MPa. The uniaxial pressure was increased to the maximum at a rate of 1 MPa/min from the moment the compact temperature reached 900 °C. To remove oxygen vacancies and possible impurities of non-oxidized carbon, the ceramics were additionally air-annealed at 1100 °C for 5 h in a muffle furnace. The resulting ceramics were then ground and polished on both sides using diamond suspensions to a thickness of 1.5 mm.

The X-ray diffraction (XRD) patterns of the prepared powders and ceramics were recorded at room temperature in the range of 20 to 80° (2θ) with a step of 0.04° and an exposure time of 2 s on an XRD-7000 powder diffractometer (Shimadzu, Kyoto, Japan) using filtered CuK_α_ radiation. Quantitative phase analysis was performed by the Rietveld method using TOPAS-Academic software, with an inaccuracy of the measurement of 1%. Original PDF-cards and CIF files of the identified phases were taken from the PDF-2 (ICDD, 2012) and the ICSD (2015) databases. The powders were calcined at a temperature of 800–1200 °C in air in a muffle furnace at a heating rate of 10 °C/min and held at a maximum temperature of 60 min to determine the effect of additional heat treatment on the crystal structure of the material.

The theoretical density of the composite phases was calculated from the results of XRD analysis according to the formula:

(1) $$\rho_{XRD}=\frac{Z\times M}{V\times N_{A}}$$

where *Z* is the number of structural units in the unit cell (16 for the cubic crystal structure of bixbyite, 4 for magnesia), *M* is an average molar mass, *V* is a unit cell volume, and *N_A_* is the Avogadro number. The theoretical density of the composites was calculated using the additive method.

The infrared spectra of the ceramics were recorded using a FT-801 IR-Fourier spectrometer (SIMEKS, Nizhny Novgorod, Russia). The density of the ceramics (*ρ*) was measured using hydrostatic weighing in distilled water on a Sartorius CPA balance (Sartorius, Göttingen, Germany) with an accuracy of 0.005 g/cm^3^, which is ~0.1% of the theoretical density. The morphology of the synthesized powders and the microstructure of the ceramics were studied using an Auriga CrossBeam scanning electron microscope (SEM) (Carl Zeiss, Jena, Germany) at an accelerating beam voltage of EHT = 3 keV with a secondary electron detector. The average grain size was estimated by measuring the width of at least 200 grains.

The emission analysis of the Er:Lu_2_O_3_-MgO and Er:Sc_2_O_3_-MgO ceramics was performed in the visible and IR range using a SOLAR M833 monochromator equipped with a Thorlabs PDA36A silicon photodetector and a PDA30G PbS fixed gain detector. Luminescence was studied in the 500–1100 nm and 1300–3000 nm ranges under excitation by CW laser diodes at wavelengths of 410 nm and 975 nm, respectively. Spatial filtering, silicon, and germanium plates were used to isolate the required spectral range of radiation. The fluorescence lifetimes for the ^4^I_11/2_ → ^4^I_13/2_ and ^4^I_13/2_ → ^4^I_15/2_ transitions were measured using a pulsed laser diode at 975 nm and a PDA20H fixed gain detector connected to an oscilloscope. Due to the strong reabsorption inside the ceramics and the noticeable effect of stimulated emission, the luminescence lifetime was measured using a modified pinhole method [25]. The pump radiation spot on the studied sample did not exceed 1 mm in diameter.

The microhardness (*HV*) of the ceramics was determined on a Qness Q60 microhardness tester (Qness, Golling an der Salzach, Austria) by measuring the lengths of the diagonals of the indentation marks on the polished sample surface under a load of 2 kg/mm^2^. A diamond pyramid with an apex angle of 136° and a diagonal length of 500 µm was selected as the indenter. The loading time was 30 s. The fracture toughness of ceramics (*K_IC_*) was calculated using the Palmquist method:

(2) $$K_{IC}=0.016\left(\frac{P}{\sqrt{c^{3}}}\right)\cdot \left(\frac{E}{\sqrt{HV}}\right)$$

where *P* is the load, *c* is the distance from the center of the print to the tip of the crack, *HV* is microhardness, and *E* is Young’s modulus. The Young’s modulus of the ceramic was assumed to be the same for the Y_2_O_3_-MgO ceramic according to [4] *E* = 240 GPa.

## 3. Results and Discussion

### 3.1. Powders Crystalline Structure and Morphology

To establish the phase composition of the selected materials, depending on the heat treatment conditions, an X-ray diffraction analysis of SHS products was carried out with dependence upon the annealing temperature.

**Figure 1:**
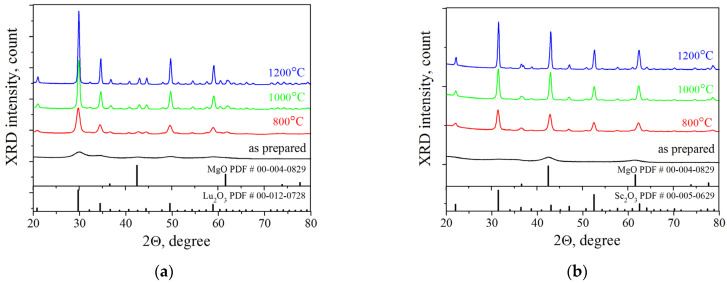
XRD patterns of the SHS-derived powders calcined at different temperatures: (**a**) 1% Er:Lu_2_O_3_-MgO; (**b**) 1% Er:Sc_2_O_3_-MgO.

Figure 1 shows the diffraction patterns of the 1% Er:Lu_2_O_3_-MgO and 1% Er:Sc_2_O_3_-MgO powders calcined at different temperatures. The as-prepared powders are almost amorphous. Calcination at a temperature of 800 °C leads to the crystallization of the powders; a mixture of cubic crystalline phases of scandia or lutetia (space group *Ia*$\bar{3}$) and magnesia (space group *Fm-3m*) is clearly seen. The integral broadening of the diffraction peaks decreases monotonically with an increase in the annealing temperature of the powder, indicating an increase in the XRD crystallite sizes. No peaks of other phases were observed in the diffraction patterns.

Characteristics of the SHS powders’ crystal structure, calculated from the results of XRD analysis, are given in Table 1. Due to amorphization, the result of the unit cell parameters calculation in the as-prepared powders shows values that differ sharply from those of the other powders. This may be due to both a systematic error in the numerical refinement because of a large integral broadening and a real increase in the unit cell parameters as a result of the size effect.

In the case of calcined powders, the calculated unit cell parameters and, hence, the theoretical phase densities, are within the confidence interval of the literature data for MgO [26], Lu_2_O_3_ [27], and Sc_2_O_3_ [28]. The addition of Er_2_O_3_ has no noticeable effect on the cell parameters due to the low concentration. It is assumed that the erbia is completely dissolved in the lutetia or scandia. The quantitative phase analysis of the calcined powders shows compliance with the specified ratio (50/50 by volume) to an accuracy of no less than 3%.

**Figure 2:**
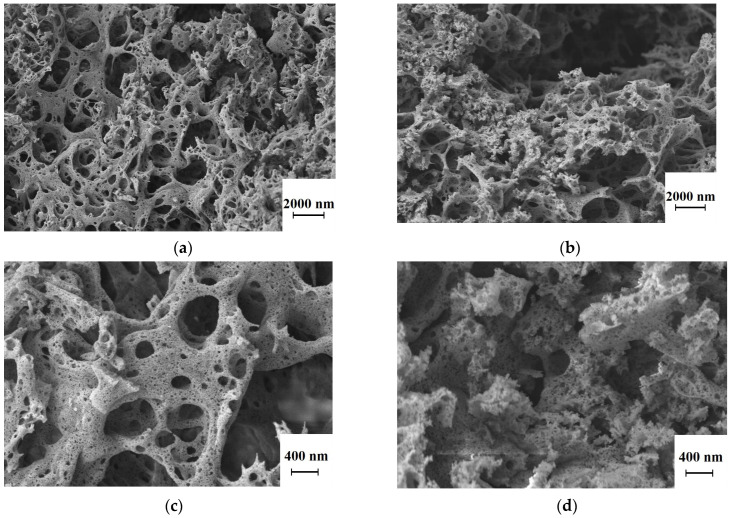
SEM images of as-synthesized powders at different magnifications: (**a**,**c**) 1% Er:Lu_2_O_3_-MgO; (**b**,**d**) 1% Er:Sc_2_O_3_-MgO.

SEM images of the synthesized powders are shown in Figure 2. The morphology of the powders is characterized by the presence of flakes with a porous structure, probably due to the release of a large amount of gaseous products during the combustion of the precursors. The characteristic size of the agglomerates is greater than 10 µm, as shown in Figure 2a,b. At a higher magnification (Figure 2b,d), the fine structure of the scales can be seen. These are fragile hollow spheres whose walls are easily destroyed by a slight impact, and the primary particles are the walls of spheres several nanometers thick and up to hundreds of nanometers in size. Such a structure is quite typical for powders prepared by glycine–nitrate SHS and is due to the synthesis mechanism. The propagation of the reaction front causes foaming of the precursor, followed by the onset of the combustion reaction. The release of a large amount of gaseous products during the reaction prevents the collapse of these pores and the rigid agglomeration of primary particles. For example, rare earth oxide powders [29,30] with a similar structure were obtained earlier.

### 3.2. Crystal Structure of Ceramics

**Figure 3:**
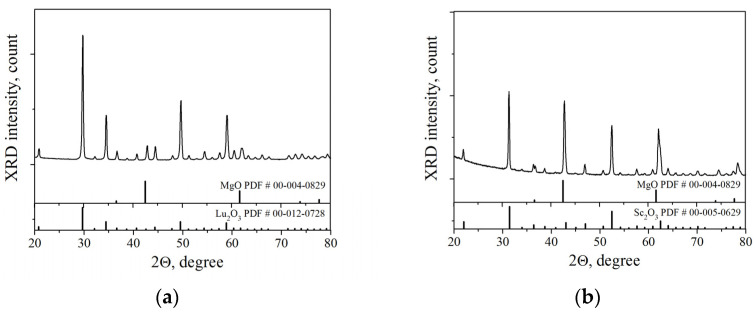
XRD patterns of the ceramics sintered at 1400 °C: (**a**) 1% Er:Lu_2_O_3_-MgO; (**b**) 1% Er:Sc_2_O_3_-MgO.

To ensure that there is no chemical interaction between the components under sintering conditions, an XRD analysis of the ceramics obtained by hot pressing at 1400 °C was performed. As can be seen from Figure 3, ceramics retain the two–phase structure of magnesia—RE sesquioxide. The intensity of the peaks in the 1% Er:Lu_2_O_3_-MgO diffraction pattern is much greater, apparently due to the more intense scattering of X-rays by the heavy lutetium atoms. The X-ray diffraction results also indicate that neither erbia nor lutetia form solid solutions with magnesia. However, it is difficult to draw a similar conclusion for the 1% Er:Sc_2_O_3_-MgO composite. For example, when 30 mol% of magnesia dissolves in scandia, the theoretical unit cell parameter changes by only 0.005 Å (from *a* = 9.846 Å to *a* = 9.841 Å) [20], which is very close to the measurement error. In Table 2 the calculated cell parameters and mass fractions of phases in the composites hot pressed at 1300 °C and 1400 °C are listed. The higher calculated fraction of MgO in the ceramic samples compared to the initial powders may indicate a systematic measurement error.

### 3.3. Microstructure and Mechanical Properties of Ceramics

The measured *HV_2_* microhardness of the obtained ceramics is in agreement with the literature data for Y_2_O_3_-MgO ceramics with grain sizes of about 200 nm [23] and is 10.4 GPa and 10.3 GPa for 1% Er:Lu_2_O_3_-MgO and 1% Er:Sc_2_O_3_-MgO composites, respectively. The crack resistance *K_IC_*, calculated using the Palmquist method, is 1.2 MPa·m^½^ for the 1% Er:Lu_2_O_3_-MgO composite and slightly higher—1.4 MPa·m^½^—for the 1% Er:Sc_2_O_3_-MgO composite. The values obtained demonstrate the high mechanical properties of the composites, which is not mandatory but very desirable for laser applications.

**Figure 4:**
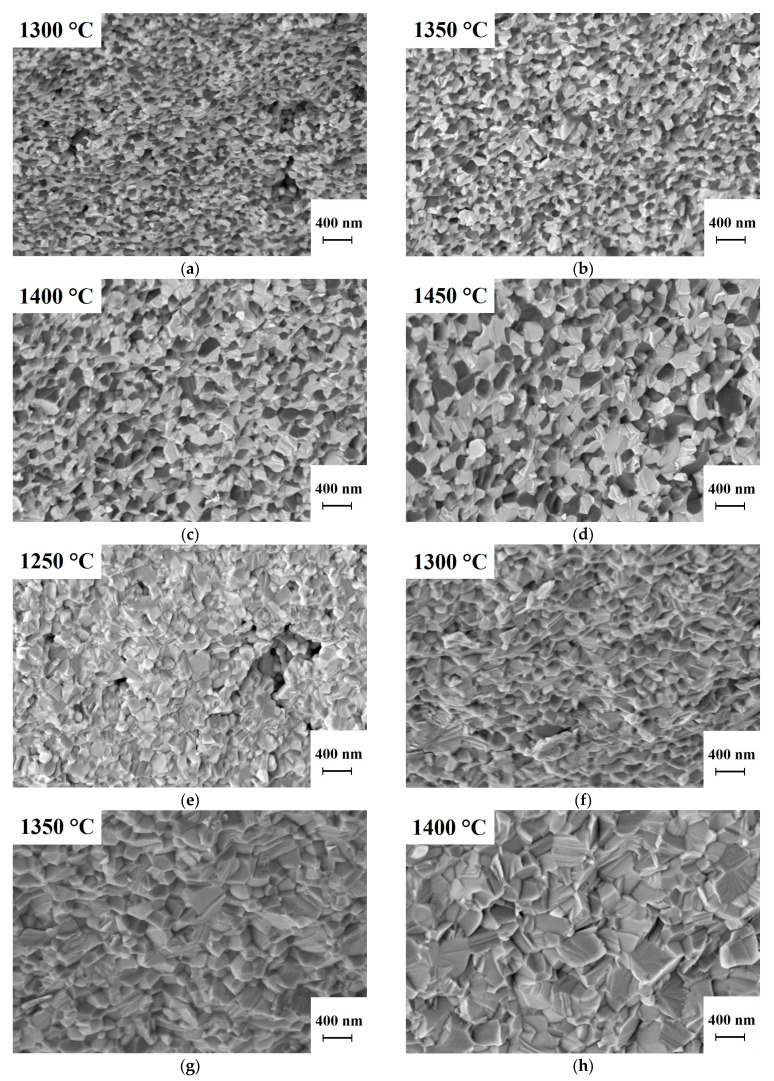
SEM images of the fractured surface of composites hot pressed at different temperatures: (**a**–**d**) 1% Er:Lu_2_O_3_-MgO; (**e**–**h**)1% Er:Sc_2_O_3_-MgO.

Figure 4 shows micrographs of fractured surfaces of ceramics 1% Er:Lu_2_O_3_-MgO and 1% Er:Sc_2_O_3_-MgO hot pressed at different temperatures. In the 1% Er:Lu_2_O_3_-MgO composite, the light “heavy” grains of the Er:Lu_2_O_3_ solid solution are clearly distinguished from the dark “light” grains of magnesia. In the 1% Er:Sc_2_O_3_-MgO composite, it is impossible to distinguish the phases, both because of the proximity of the effective atomic masses of Sc_2_O_3_ and MgO and because of their averaging due to the possible formation of solid solutions.

The lutetia-based composite sinters at temperatures similar to Y_2_O_3_-MgO [31]. The 1% Er:Lu_2_O_3_-MgO sample hot pressed at 1300 °C has pores much larger than ceramic grains. As the temperature increases, the porosity decreases significantly and only isolated pores less than 100 nm in diameter are found. At 1400 °C, a dense ceramic structure is formed; further increase in hot pressing temperature is impractical as it only increases the average grain size without having any effect on the porosity. Table 3 summarizes the densities and average grain size of the composites at different hot-pressing temperatures. In 1% Er:Sc_2_O_3_-MgO ceramics, grain growth is much more intense with increasing hot-pressing temperatures, probably due to the greater mutual solubility of Sc_2_O_3_ and MgO and, consequently, the greater diffusion rate. Although the fractograms show that the pore-free structure of the 1% Er:Sc_2_O_3_-MgO ceramic is only achieved at a hot-pressing temperature of 1350 °C, as will be shown below, the optimum temperature in terms of optical transparency is 50 °C lower.

### 3.4. Transmittance and Luminescence of Er^3+^ Ions in Composite Ceramics

**Figure 5:**
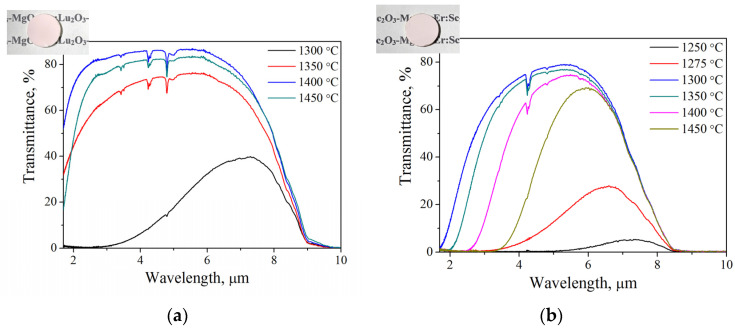
IR Fourier transmission spectra of composite ceramics sintered at different temperatures: (**a**) 1% Er:Lu_2_O_3_-MgO and (**b**) 1% Er:Sc_2_O_3_-MgO. Inserts are the appearance of samples sintered at 1400 °C.

Such a difference in microstructure determines the difference in the transmission level of 1% Er:Lu_2_O_3_-MgO and 1% Er:Sc_2_O_3_-MgO ceramics. Figure 5 shows the IR range transmission spectra of the composites obtained by hot pressing at different temperatures. A decrease in porosity with increasing hot-pressing temperature leads to a decrease in scattering at the pores; however, at the same time, the average grain size increases, which increases the Fresnel reflection loss at the phase boundaries. The 1% Er:Sc_2_O_3_-MgO ceramics have a shallow absorption edge at short wavelengths; the highest maximum transmission is shown by a sample sintered at a temperature of 1300 °C, which achieves the optimal ratio of minimum porosity to minimum grain size. The 1% Er:Lu_2_O_3_-MgO composites have higher optical properties over the entire measurement range. The sample obtained at 1400 °C at a wavelength of 1.5 μm has a transmission of ~60%, and in the range of 3–6 μm its transmission corresponds to a lutetia single crystal. The observed absorption bands in the spectra are associated with atmospheric water and carbon dioxide, as well as carbon-containing compounds trapped in the pores and formed as a result of the interaction of the carbon mold with the composites.

**Figure 6:**
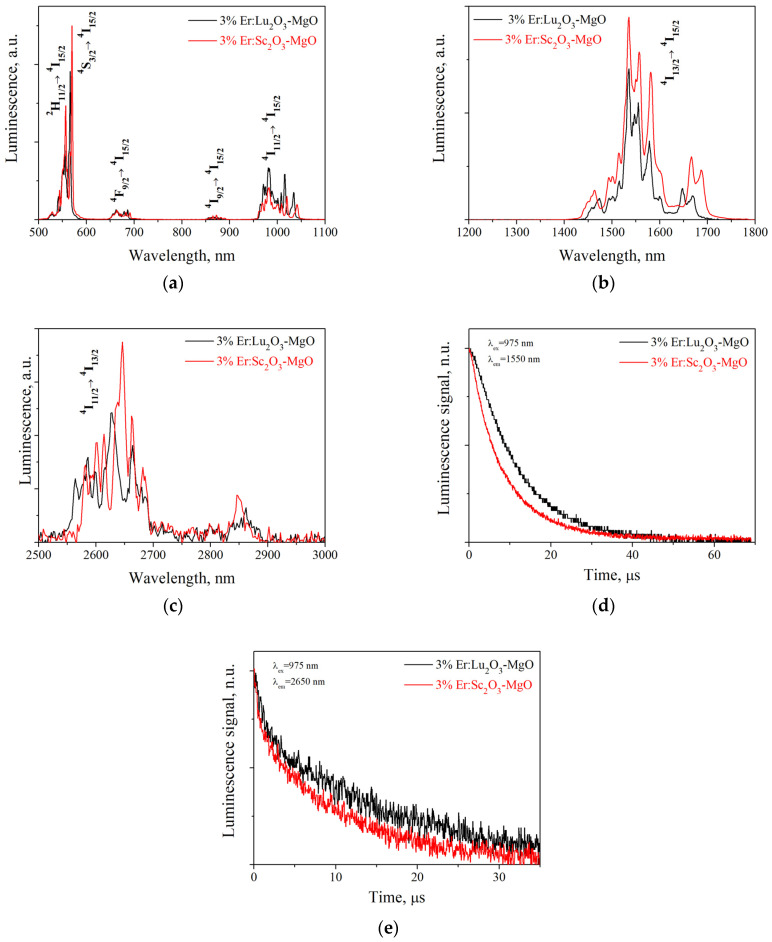
Luminescence of 3% Er:Lu_2_O_3_-MgO and 3% Er:Sc_2_O_3_-MgO ceramics: (**a**) In the visible range under 410 nm excitation; (**b**) In the near-IR regions under 975 nm excitation; (**c**) In the mid-IR regions under 975 nm excitation; (**d**,**e**) Luminescence decay curves for the ^4^I_13/2_ and ^4^I_11/2_ state measured at 1 mm pinhole.

Figure 6 shows the results of a luminescence study of the 3% Er:Lu_2_O_3_-MgO and 3% Er:Sc_2_O_3_-MgO ceramics in the visible and IR regions. Note that in addition to erbium ions, luminescence in magnesia-based materials can also be associated with F-type centers in the crystal lattice [32,33], but they require UV or X-rays to be excited. Therefore, this luminescence mechanism is not considered. Both ceramic samples exhibit a strong green luminescence band under 410 nm excitation (Figure 6a), as well as a noticeable upconversion luminescence (associated with ^4^F_9/2_ → ^4^I_15/2_ and ^2^H_11/2_/^4^S_3/2_ → ^4^I_15/2_ transitions) in the same region when pumped at 975 nm, which is characteristic of Er^3+^ ions [34]. A feature of the near-IR luminescence is the presence of long-wavelength spectral components around 1650 and 1670 nm for 3% Er:Lu_2_O_3_-MgO and 3% Er:Sc_2_O_3_-MgO ceramics, respectively (Figure 6b). The measured lifetime values of the ^4^I_13/2_ state are 8.85 ± 0.1 ms for 3% Er:Lu_2_O_3_-MgO and 5.7 ± 0.2 ms for 3% Er:Sc_2_O_3_-MgO ceramics (Figure 6d), which are slightly longer than the typical values of Er^3+^ ion lifetime in oxide matrices [17].

The higher luminescence lifetime in Lu_2_O_3_-MgO composite could be explained by the difference in maximum phonon energy for Er:Lu_2_O_3_ (611 cm^−1^) and for Er:Sc_2_O_3_ (666 cm^−1^) [35]. The higher maximum phonon energy of Er:Sc_2_O_3_ implies a higher probability of non-radiative multiphonon relaxation, which decreases the decay lifetime, especially in the long wavelength region.

The mid-IR emission was almost identical for both ceramic samples and was represented by broad bands at 2650 and 2850 nm (Figure 6c). The mid-IR luminescence decay curves were well approximated by double exponential functions (Figure 6e). The corresponding fast decay constants are 1.5 ± 0.1 ms for 3% Er:Lu_2_O_3_-MgO and 0.7 ± 0.15 ms for 3% Er:Sc_2_O_3_-MgO ceramics, which is close to the lifetime of ~2.8 μm luminescence in Er:Lu_2_O_3_ ceramics [17].

This study is the first to demonstrate the intense luminescence of erbium ions in RE_2_O_3_-MgO composite ceramics in the mid-IR region. Additionally, although further comparative studies with Y_2_O_3_-MgO composites are needed to select the optimal matrix, the obtained results indicate the potential for using the developed materials in laser technology.

## 4. Conclusions

The SHS method was successfully applied to obtain highly dispersed powders of 1% Er:Lu_2_O_3_-MgO and 1% Er:Sc_2_O_3_-MgO. The prepared powders had a two-phase structure and morphology in the form of sponge-like agglomerates. Hot pressing of scandia-based ceramics occurs at lower temperatures, but with more intense grain growth than that of lutetia-based ceramics. The highest transmittances of 1.5 mm thick composites are achieved 84.5% and 78.9% in the IR range for Er:Lu_2_O_3_-MgO and Er:Sc_2_O_3_-MgO hot pressed at 1400 °C and 1300 °C, respectively. The strong luminescence of erbium ions in the range of ~1.6 and ~2.6–2.8 μm is shown to have sufficient lifetime values (8.85 ± 0.1 ms for 1% Er:Lu_2_O_3_-MgO and 5.7 ± 0.2 ms for 1% Er:Sc_2_O_3_-MgO ceramics of the ^4^I_13/2_ state) to allow the composites to be considered for use in laser technology.

## Figures and Tables

**Table 1:** Mass fractions in the synthesis product calculated from the XRD results: lattice parameters *a*, theoretical densities *ρ_XRD_* of the rare earth sesquioxides and magnesia in the prepared powders at different calcination temperatures, and the volume fraction of magnesia.

**1% Er:Lu_2_O_3_-MgO**							
**Calcination Temperature**	**Mass Fraction (Lu_2_O_3_), % wt.**	**Mass Fraction (MgO), % wt.**	***a* (Lu_2_O_3_), Å**	***a* (MgO), Å**	***ρ_XRD_* (Lu_2_O_3_), g/cm^3^**	***ρ_XRD_* (MgO), g/cm^3^**	**Volume Fraction (MgO), %**
As prepared	69.7	30.3	10.53 (9)	4.29 (4)	9.07	3.38	53.8
800 °C	72.0	28.0	10.398 (8)	4.228 (4)	9.40	3.54	50.8
1000 °C	73.2	26.8	10.399 (3)	4.221 (2)	9.40	3.56	49.2
1200 °C	74.0	26.0	10.392 (2)	4.217 (2)	9.42	3.57	48.1
**1% Er:Sc_2_O_3_-MgO**							
**Calcination Temperature**	**Mass Fraction (Sc_2_O_3_), % wt.**	**Mass Fraction (MgO), % wt.**	***a* (Sc_2_O_3_), Å**	***a* (MgO), Å**	***ρ_XRD_* (Sc_2_O_3_), g/cm^3^**	***ρ_XRD_* (MgO), g/cm^3^**	**Volume Fraction (MgO), %**
As prepared	58.8	41.2	9.6 (2)	4.30 (9)	4.17	3.37	46.5
800 °C	54.3	45.7	9.857 (9)	4.223 (4)	3.83	3.55	47.6
1000 °C	54.6	45.4	9.856 (6)	4.219 (3)	3.83	3.56	47.2
1200 °C	53.7	46.3	9.858 (3)	4.218 (2)	3.82	3.57	48.0

**Table 2:** Mass fractions in the 1% Er:Lu_2_O_3_-MgO and 1% Er:Sc_2_O_3_-MgO composites calculated from the XRD results: lattice parameters *a*, theoretical densities *ρ_XRD_* of the rare earths sesquioxides and magnesia, and the volume fraction of magnesia.

**1% Er:Lu_2_O_3_-MgO**							
**Hot Pressing Temperature, °C**	**Mass Fraction (Lu_2_O_3_), % wt.**	**Mass Fraction (MgO), % wt.**	***a* (Lu_2_O_3_), Å**	***a* (MgO), Å**	***ρ_XRD_* (Lu_2_O_3_), g/cm^3^**	***ρ_XRD_* (MgO), g/cm^3^**	**Volume Fraction (MgO), %**
1300	68	32	10.374 (3)	4.217 (1)	9.47	3.57	56
1400	69	31	10.375 (3)	4.216 (1)	9.48	3.57	54
**1% Er:Sc_2_O_3_-MgO**							
**Hot Pressing Temperature, °C**	**Mass Fraction (Sc_2_O_3_), % wt.**	**Mass Fraction (MgO), % wt.**	***a* (Sc_2_O_3_), Å**	***a* (MgO), Å**	***ρ_XRD_* (Sc_2_O_3_), g/cm^3^**	***ρ_XRD_* (MgO), g/cm^3^**	**Volume Fraction (MgO), %**
1300	46	54	9.838 (2)	4.217 (2)	3.85	3.57	56
1400	46	54	9.836 (2)	4.217 (2)	3.85	3.57	56

**Table 3:** Average grain size and relative density of the 1% Er:Lu_2_O_3_-MgO and 1% Er:Sc_2_O_3_-MgO composites hot pressed at different temperatures.

Hot Pressing Temperature, °C	1% Er:Lu_2_O_3_-MgO		1% Er:Sc_2_O_3_-MgO	
	Average Grain Size, nm	Relative Density, %	Average Grain Size, nm	Relative Density, %
1250			152	94.1
1275			158	97.5
1300	112	96.9	189	>99.5
1350	151	>99.5	235	>99.5
1400	176	>99.5	319	>99.5
1450	185	>99.5	559	>99.5

## Data Availability

Not applicable.
